# Supplementary material for: Malaria prevalence and incidence in an isolated, meso-endemic area of Mozambique
Source: PeerJ. 2015 Nov 5;3:e1370. doi: 10.7717/peerj.1370 (PMC4647567; doi:10.7717/peerj.1370)

**Linga Linga Malaria Analysis**

Description of the data

There were three main sources of data:

1. Household-level census data (2007)
2. GPS coordinates of surveyed households
3. Parasitology dataset (2007-2011)
4. Overview of the census data

The 2007 census data contained information at the household level on 482 households (479 unique house numbers), and information on the individual level on 983 people (from 475 unique households).

Household information includes the following:

- Length of time they’ve lived in the house
- Number of people living in the house
- Source of drinking water
- Source of water for washing
- Whether or not they own any animals
- Number of bedrooms
- Number of beds
- Type of roof
- Type of walls
- Length of opening between roof and walls
- Whether or not they own a radio, mobile phone, bicycle or boat

Additional individual-level information includes the age, sex, bed net ownership and education level of the individual.

1. Overview of parasitology datasets

The parasitology datasets contained information on the individual’s house number, name (except for 2008 and 2010), age, sex, whether or not they tested positive for malaria, and whether or not they used a bed net the previous night (except for 2008).

A large proportion of individuals tested had missing house number information, and as such could not be matched with the census data. The proportion missing increased with each successive year. An overview of the parasitology datasets, including the number of individuals per dataset, and the number of individuals that could be matched with the 2007 census data, is presented in Table 1. In comparing the proportion of positive individuals in the raw dataset with those in the matched data, 2007, 2009, 2010 and 2011 were very similar, whereas the prevalence was much higher in the raw dataset in 2008. In order to explore this a little more closely, raw data prevalence, plus the age and sex of the individuals was summarised according to whether or not the individual had a house number for 2008 (Table 1).

| **Variable** | | **Completed house number** | | | **Missing house number** | |
| --- | --- | --- | --- | --- | --- | --- |
|  |  | N | (%) | N | | (%) |
| **Malaria** | **Positive** | 51 | (25%) | 66 | | (47%) |
|  | **Negative** | 153 | (75%) | 75 | | (53%) |
|  |  |  |  |  | |  |
| **Age group** | **< 1** | 3 | ( 1%) | 1 | | ( 1%) |
|  | **1-4** | 8 | ( 4%) | 5 | | ( 4%) |
|  | **5-9** | 41 | (20%) | 31 | | (22%) |
|  | **10-15** | 57 | (28%) | 26 | | (18%) |
|  | **16-25** | 10 | ( 5%) | 9 | | ( 6%) |
|  | **>25** | 41 | (20%) | 14 | | (10%) |
|  | **Missing** | 44 | (22%) | 55 | | (39%) |
|  |  |  |  |  | |  |
| **Sex** | **Male** | 120 | (59%) | 68 | | (48%) |
|  | **Female** | 69 | (34%) | 48 | | (34%) |
|  | **Missing** | 15 | ( 7%) | 25 | | (18%) |

Table 1: Summary of characteristics of individuals according to whether or not they had been allocated a house number for 2008 parasitology survey)


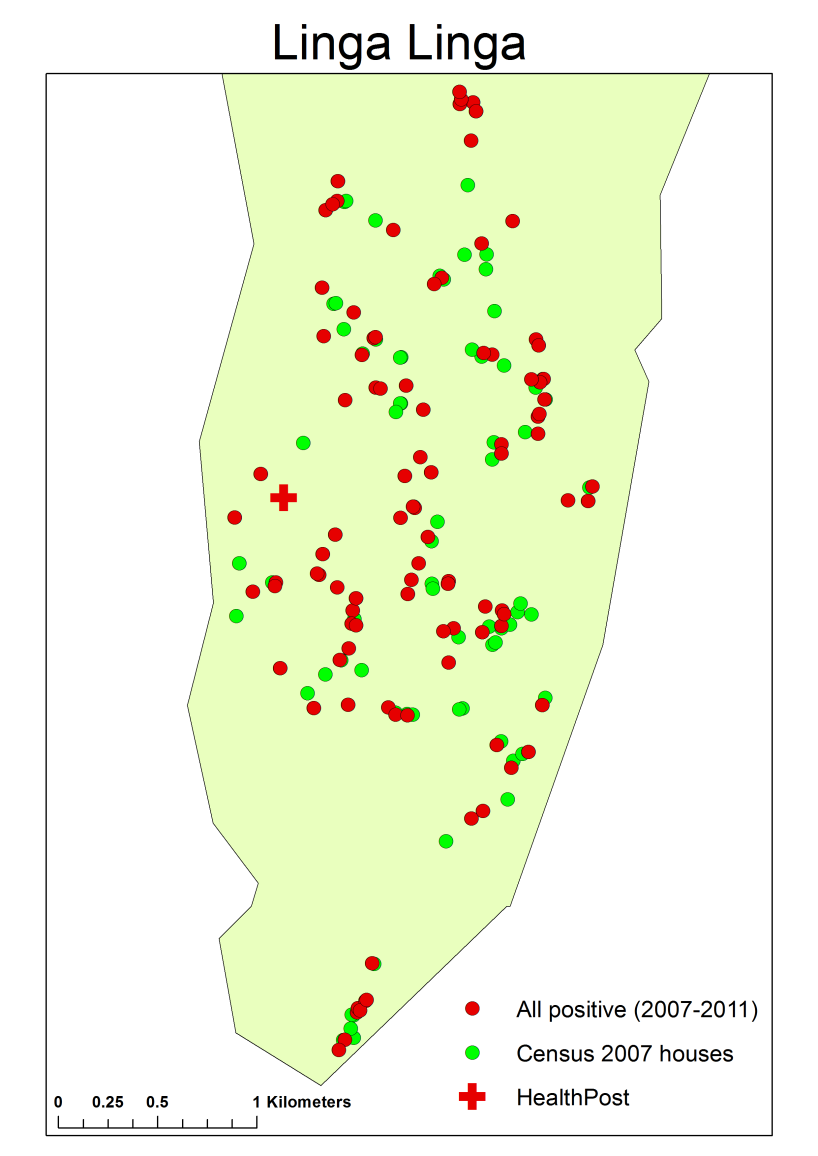


*Figure 1: Locations of positive cases between 2007-20011*

|  | **OR** | **95% CI** | **p-value** |
| --- | --- | --- | --- |
|  |  |  |  |
| **Year** |  |  |  |
| ***2007*** | - |  |  |
| ***2008*** | 2.1463 | (1.1236, 4.0463) | 0.0190 |
| ***2009*** | 12.1932 | (7.1587, 21.2718) | <0.0001 |
| ***2010*** | 3.6402 | (2.0217, 6.5814) | <0.0001 |
| ***2011*** | 6.2109 | (3.1586, 12.3187) | <0.0001 |
|  |  |  |  |
| **Sex** |  |  |  |
| ***Female*** | - |  |  |
| ***Male*** | 1.4818 | (0.9866, 2.2278) | 0.0581 |
|  |  |  |  |
| **Age group** |  |  |  |
| ***< 1*** | - |  |  |
| ***1-4*** | 3.0173 | (0.9157, 10.8399) | 0.0772 |
| ***5-9*** | 2.9534 | (1.0250, 9.4717) | 0.0534 |
| ***10-15*** | 2.3662 | (0.8190, 7.5887) | 0.1248 |
| ***16-25*** | 0.9304 | (0.2317, 3.7859) | 0.9184 |
| ***>25*** | 1.1246 | (0.3775, 3.67530 | 0.8378 |
|  |  |  |  |
| **No people** |  |  |  |
| ***1*** | - |  |  |
| ***2*** | 1.6918 | (0.8307, 3.5808) | 0.1564 |
| ***3*** | 2.2697 | (1.1212, 4.7798) | 0.0260 |
| ***>3*** | 0.9894 | (0.4854, 2.0845) | 0.9771 |
|  |  |  |  |
| **Wall category** |  |  |  |
| ***Other*** | - |  |  |
| ***‘Green’*** | 0.5020 | (0.2488, 1.0080) | 0.0527 |
|  |  |  |  |
| **Roof category** |  |  |  |
| ***Other*** | - |  |  |
| ***‘Green’*** | 1.6334 | (1.0056, 2.6958) | 0.0506 |
|  |  |  |  |
| **Distance** | 0.9996 | (0.9994, 0.9999) | 0.0117 |
|  |  |  |  |

Table 2: Adjusted odds-ratios obtained from fitting a multiple logistic regression model

A non-spatially structured random household-level intercept was added to the model, but as this wasn’t significant it was not included.

A spatially smooth term was added to the model, to determine whether there was any spatial pattern in the malaria cases after adjusting for the other risk factors in the model. The resulting generalised additive model (GAM) is included in the article text.


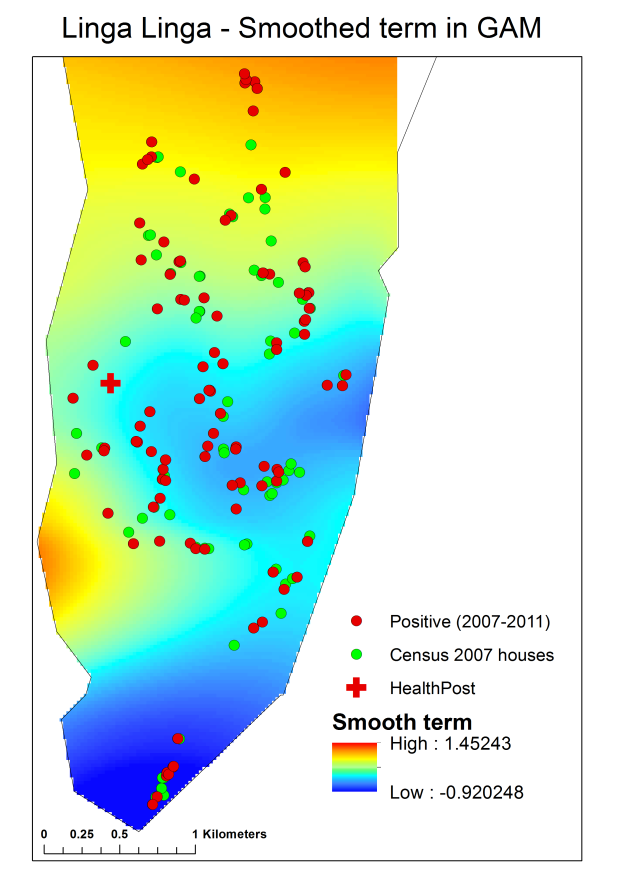

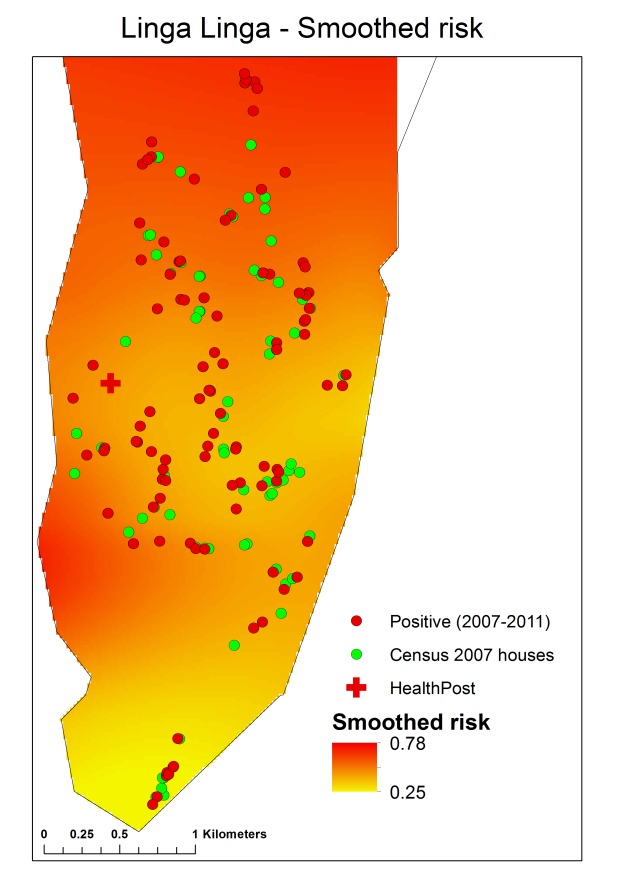


*Figure 2: Smoothed results from GAM*

Individuals who test positive for malaria more than once

Of the 220 matched entries with non-missing names, 86 people were tested more than once. Of these 86, 17 people tested positive more than once.

Of the 17, 11 were between the ages of 5 and 9 when they first tested positive. Further, 9 were male and 8 were female.

The households were in the northern half of the study area. The maps indicate the location of households with at least one positive case by year of survey.


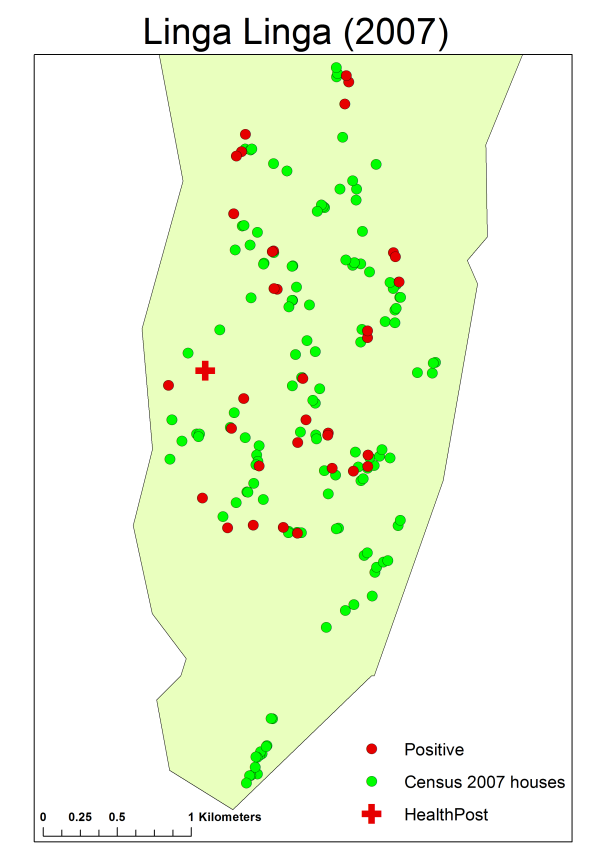

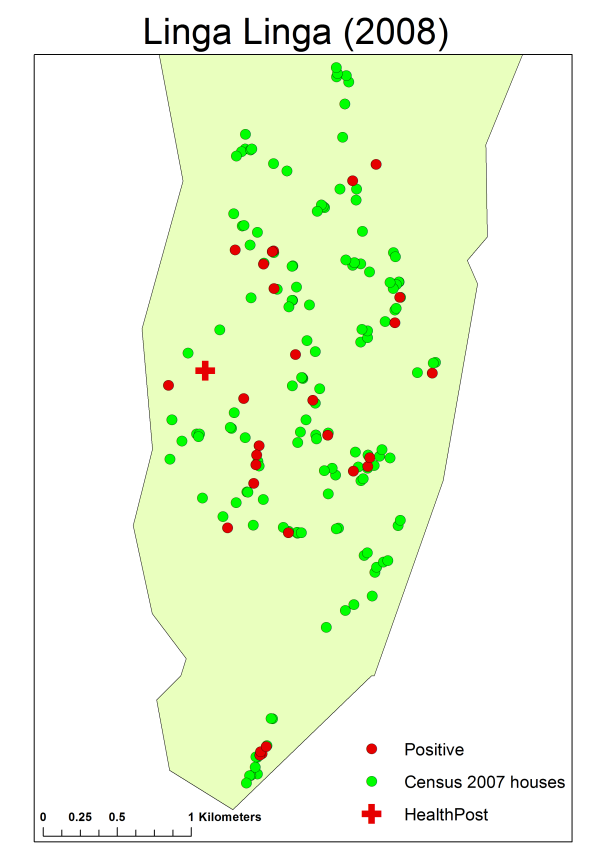

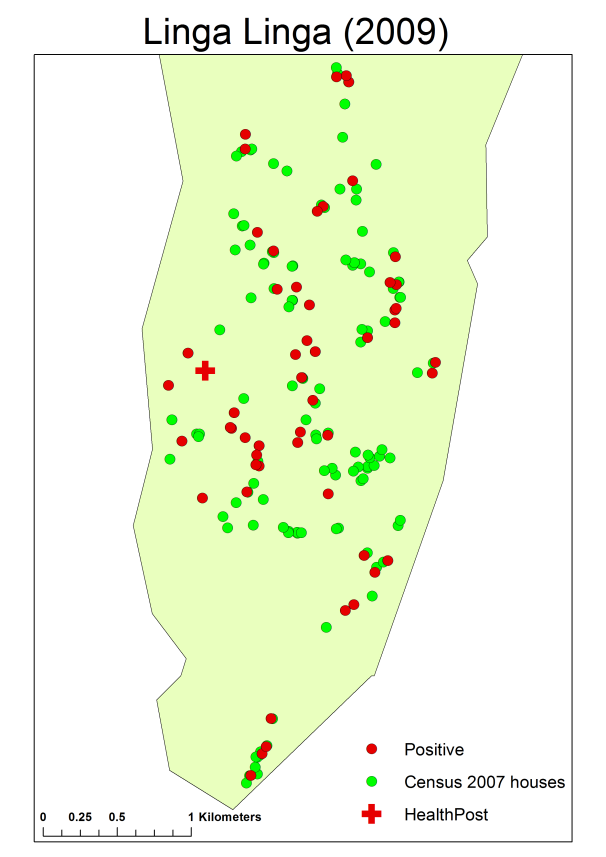

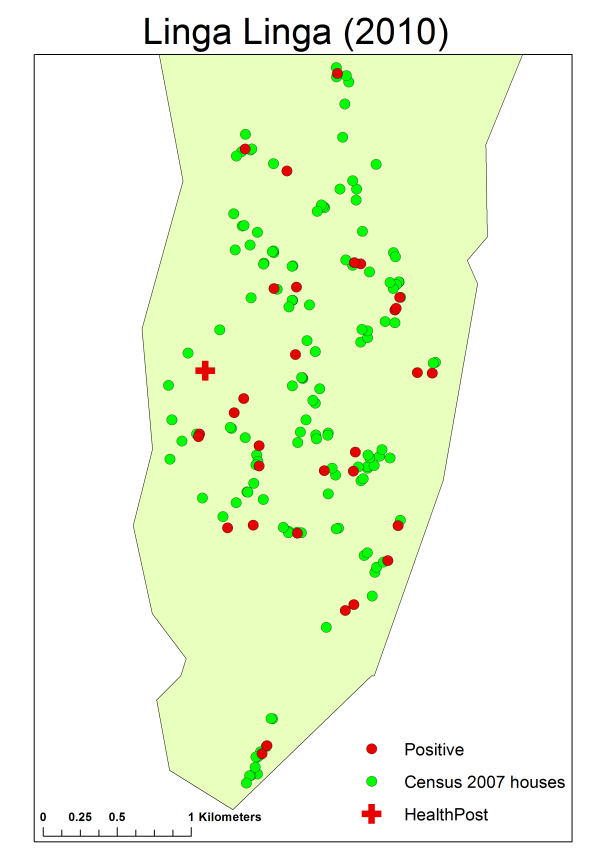

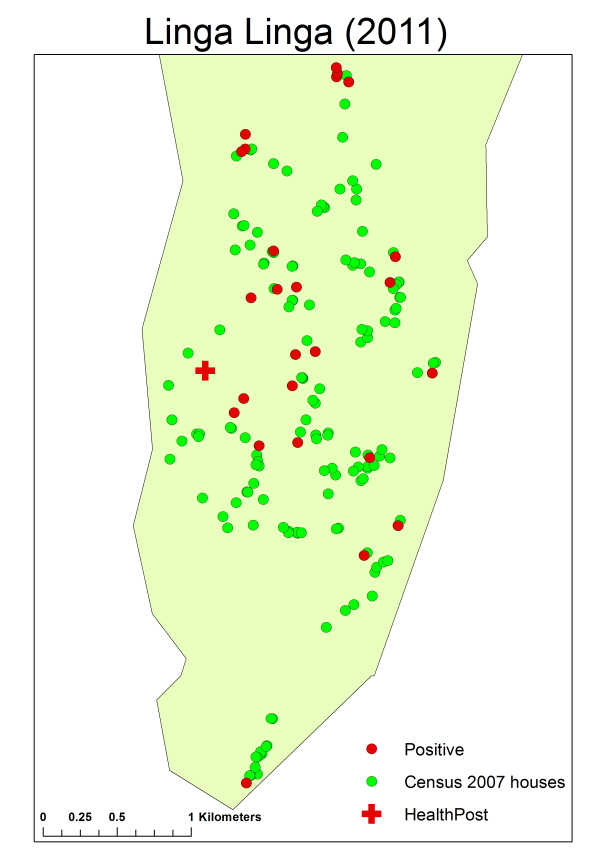

Supplement: File S1 [file peerj-03-1370-s001.docx]
