# Supplementary material for: Malaria prevalence and incidence in an isolated, meso-endemic area of Mozambique
Source: PeerJ. 2015 Nov 5;3:e1370. doi: 10.7717/peerj.1370 (PMC4647567; doi:10.7717/peerj.1370)
Supplement: File S2 [file peerj-03-1370-s002.docx]

**Health Post data**

**Summary of findings:**

**Health centre visitors**

- Over the study period there are 4949 visits to the health centre, with 87% of visitors (4321/4949) residing in Linga Linga (Table 2)
- On mapping the number of visits per household, weighted by number of people in the household (using census data), there was some evidence that those living further away from the health centre were less likely to attend (Table 5, Figures 1 & 2)
- Median age of attendance is 23 (inter-quartile range 7-44) (Table 4). It’s difficult to compare this figure to the overall population of Linga Linga due to coding issues in the individual census data, but this is potentially just reflecting the age distribution of the overall population
- More females attend the health centre than males (Table 4). Individual census data indicates that 54% of population are female, hence the health post data simply reflects this
- Majority of those attending the health centre use bed nets (80%) (Table 4)

**Clinically diagnosed with malaria**

- Of the Linga Linga residents attending the health centre, 31% (1345/4321) of visits are clinically diagnosed with malaria (Table 3)
- The clinically diagnosed population are slightly younger than those attending the health centre for all ailments (median 20, IQR 6-38) (Table 4)
- Some evidence of a relationship between number of visits clinically diagnosed with malaria per person per household and distance to health centre (Table 5)
- No significant evidence of clustering of cases
- Percentage of visits clinically diagnosed with malaria decreased slightly over time (Table 3, Figure 9)

**Confirmed malaria cases**

- Of the 1345 visits clinically diagnosed with malaria, 65% were tested (870/1345) (Table 3). There doesn’t appear to be any spatial structure to the locations from which people were tested (Figure 5).
- In looking at the proportion of clinically diagnosed cases tested over time (Figure 10) it was observed that there were large periods of time over which no cases were confirmed.
- The age of those confirmed to have malaria was young (median 12, IQR 4-30) (Table 4)
- The greatest number of positive tests occurred in the centre of Linga Linga, however it is difficult to draw any firm conclusions about the distribution of malaria in Linga Linga from this observation.
- The proportion of clinically confirmed cases that were diagnosed as positive has increased slightly over time (Table 3, Figure 11).

Table 1: Number attending the health post & malaria

|  | **2009** | | **2010** | | **2011** | | **Total** | |
| --- | --- | --- | --- | --- | --- | --- | --- | --- |
|  |  |  |  |  |  |  |  |  |
| Attended health post | 1144 |  | 2655 |  | 1150 |  | 4949 |  |
|  |  |  |  |  |  |  |  |  |
| Diagnosed with malaria | 399 | (35%) | 888 | (33%) | 271 | (24%) | 1558 | (31%) |
|  |  |  |  |  |  |  |  |  |
| Tested for malaria (% of diagnosed) | 131 | (33%) | 695 | (78%) | 186 | (68%) | 1012 | (65%) |
|  |  |  |  |  |  |  |  |  |
| Confirmed positive (% of tested) | 56 | (43%) | 423 | (61%) | 145 | (78%) | 624 | (62%) |

Table 2: Place of residence of those attending the health post

|  | **2009** | | **2010** | | **2011** | | **Total** | |
| --- | --- | --- | --- | --- | --- | --- | --- | --- |
|  |  |  |  |  |  |  |  |  |
| Attended health post | 1144 |  | 2655 |  | 1150 |  | 4949 |  |
|  |  |  |  |  |  |  |  |  |
| Linga Linga resident | 1001 | (88%) | 2330 | (88%) | 990 | (86%) | 4321 | (87%) |
|  |  |  |  |  |  |  |  |  |
| Completed house number (% of Linga Linga residents) | 579 | (58%) | 1318 | (57%) | 518 | (52%) | 2415 | (56%) |
|  |  |  |  |  |  |  |  |  |
| Unique households | 229 | - | 324 | - | 189 | - | 396 | - |
| Unique households with GPS coordinates (% of unique households) | 189 | (83%) | 261 | (81%) | 161 | (85%) | 310 | (78%) |

Table 3: Linga linga residents & malaria

|  | **2009** | | **2010** | | **2011** | | **Total** | |
| --- | --- | --- | --- | --- | --- | --- | --- | --- |
|  |  |  |  |  |  |  |  |  |
| Attended health post | 1001 |  | 2330 |  | 990 |  | 4321 |  |
|  |  |  |  |  |  |  |  |  |
| **Diagnosed with malaria** | **352** | **(35%)** | **756** | **(32%)** | **237** | **(24%)** | **1345** | **(31%)** |
| *Completed house number* | 206 | (58%) | 407 | (54%) | 131 | (56%) | 744 | (55%) |
| *Unique household* | 131 | - | 198 | - | 96 | - | 253 | - |
| *Unique household with coordinates* | 112 | (85%) | 172 | (87%) | 84 | (88%) | 211 | (83%) |
|  |  |  |  |  |  |  |  |  |
| **Tested for malaria (% of diagnosed)** | **116** | **(33%)** | **594** | **(79%)** | **160** | **(68%)** | **870** | **(65%)** |
|  |  |  |  |  |  |  |  |  |
| **Confirmed positive (% of tested)** | **51** | **(44%)** | **368** | **(62%)** | **126** | **(79%)** | **545** | **(63%)** |
| *Completed house number* | 27 | (53%) | 195 | (53%) | 69 | (55%) | 291 | (53%) |
| *Unique household* | 23 |  | 131 |  | 56 |  | 161 |  |
| *Unique household with coordinates* | 18 | (78%) | 117 | (89%) | 51 | (91%) | 139 | (86%) |

Table 4: Demographic characteristics of Linga linga residents attending the health post. Note, some people will be included more than once if there are repeat visits to the health centre

|  | **Total** | |
| --- | --- | --- |
|  |  |  |
| **Attended health post** | **4321** |  |
| Age | 23 | (7-44 IQR) |
|  |  |  |
| Sex: Male | 1769 | 41% |
| Female | 2552 | 59% |
|  |  |  |
| Nets: Yes | 3467 | 80% |
| No | 839 | 19% |
| Missing | 15 | 1% |
|  |  |  |
| **Diagnosed with malaria** | **1345** |  |
| Age | 20 | (6-38 IQR) |
| Sex: Male | 541 | 40% |
| Female | 804 | 60% |
|  |  |  |
| Nets: Yes | 1091 | 81% |
| No | 251 | 19% |
| Missing | 3 | <1% |
|  |  |  |
| **Tested for malaria (% of diagnosed)** | **870** | **(65%)** |
| Age | 20 | (6-38 IQR) |
|  |  |  |
| Sex: Male | 374 | 43% |
| Female | 496 | 57% |
|  |  |  |
| Nets: Yes | 722 | 83% |
| No | 145 | 17% |
| Missing | 3 | <1% |
|  |  |  |
| **Confirmed positive (% of tested)** | **545** | **(63%)** |
| Age | 12 | (4-30 IQR) |
|  |  |  |
| Sex: Male | 247 | 45% |
| Female | 298 | 55% |
|  |  |  |
| Nets: Yes | 452 | 83% |
| No | 92 | 17% |
| Missing | 1 | <1% |

Table 5: Correlation between distance to health post and household level variables

|  | Correlation coefficient (Spearman’s rank) | p-value |
| --- | --- | --- |
| All visits: no of visits per person per household | -0.1492 | 0.0031 |
| Clinically diagnosed malaria: no of visits per person per household | -0.1004 | 0.0473 |
| Clinically diagnosed malaria: proportion of cases tested | 0.0166 | 0.8108 |
| Confirmed malaria: no of cases | -0.1075 | 0.1566 |


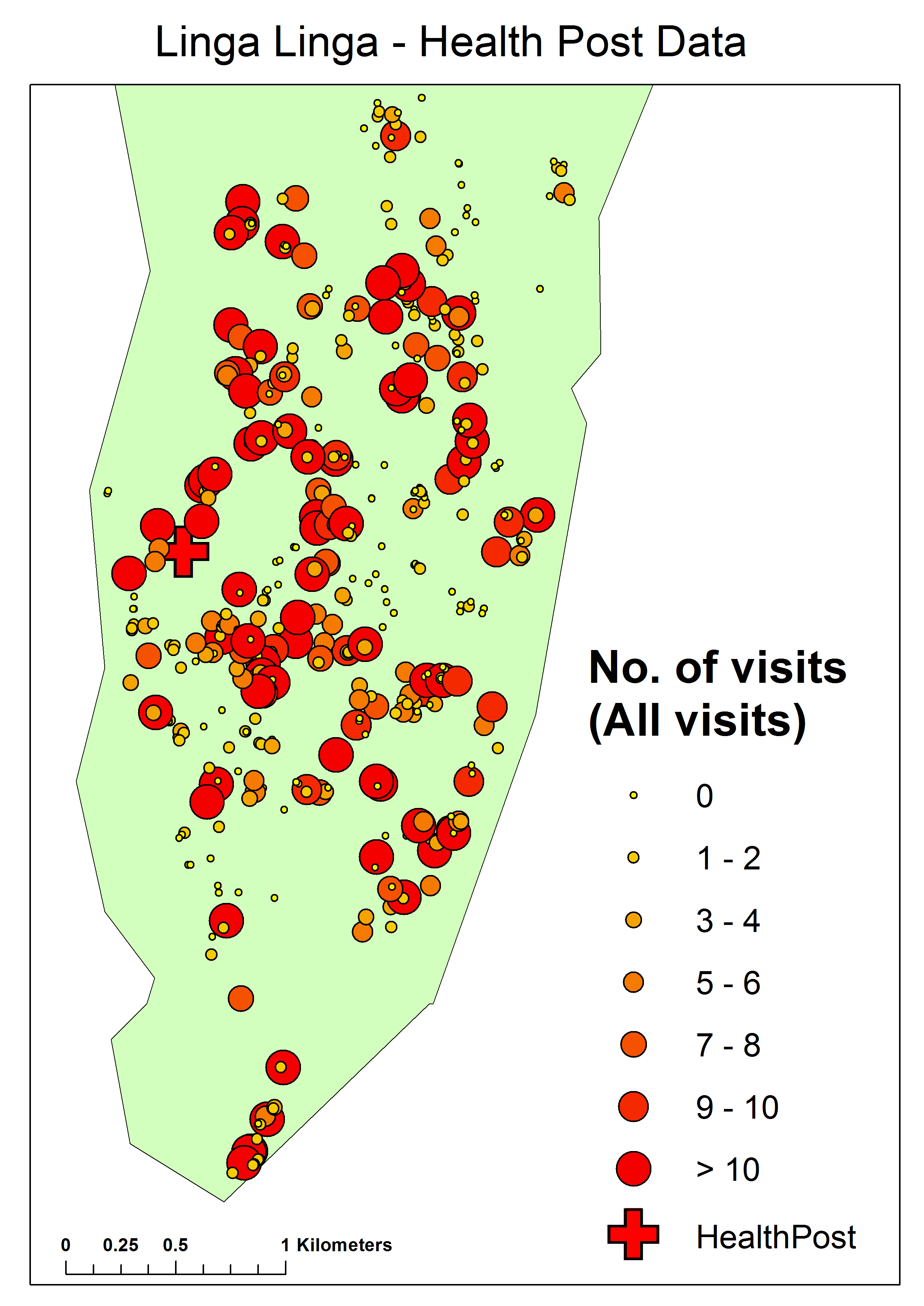


Figure 1: Number of visits per household to the health post


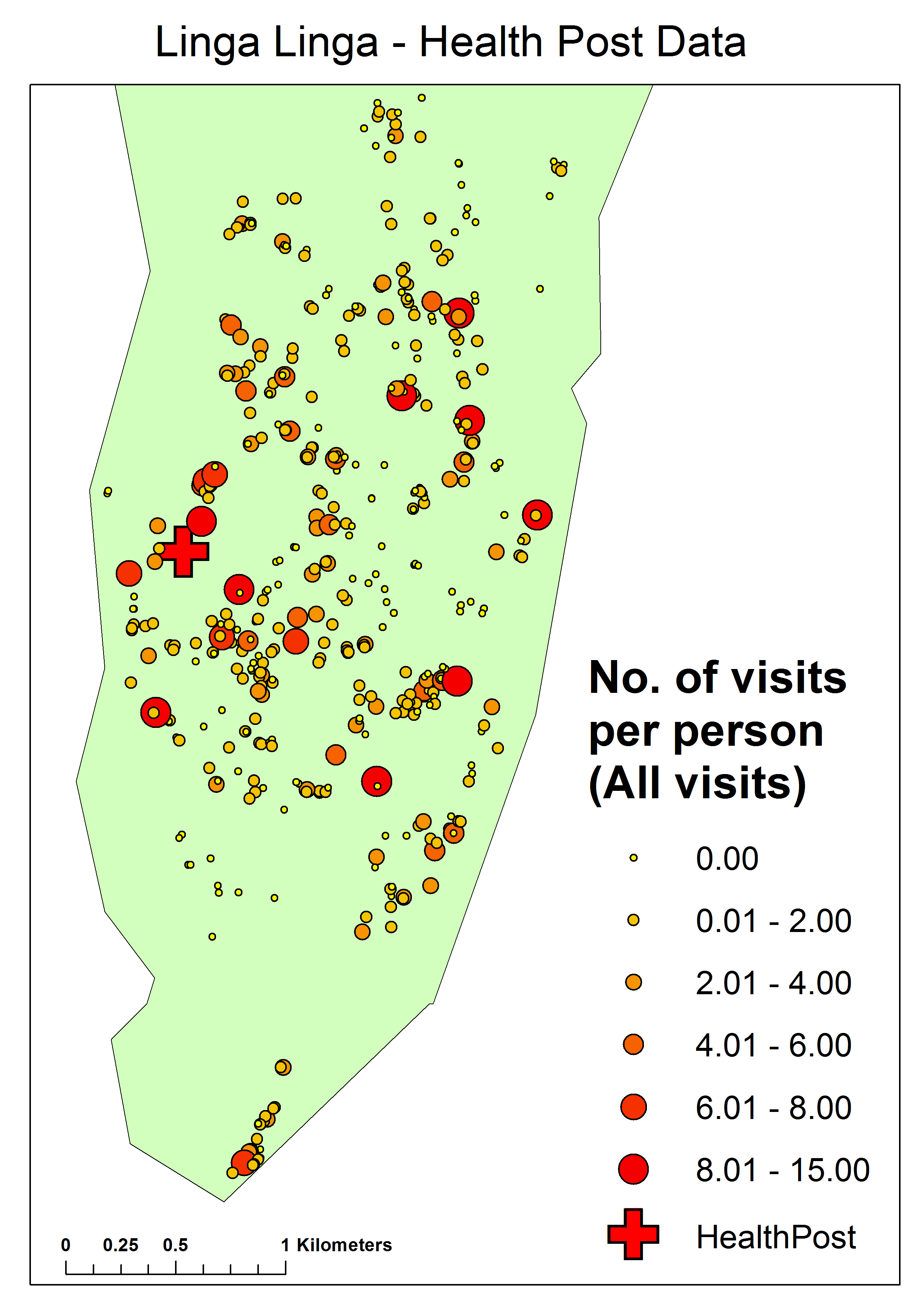


Figure 2: Number of visits per person per household to the health post


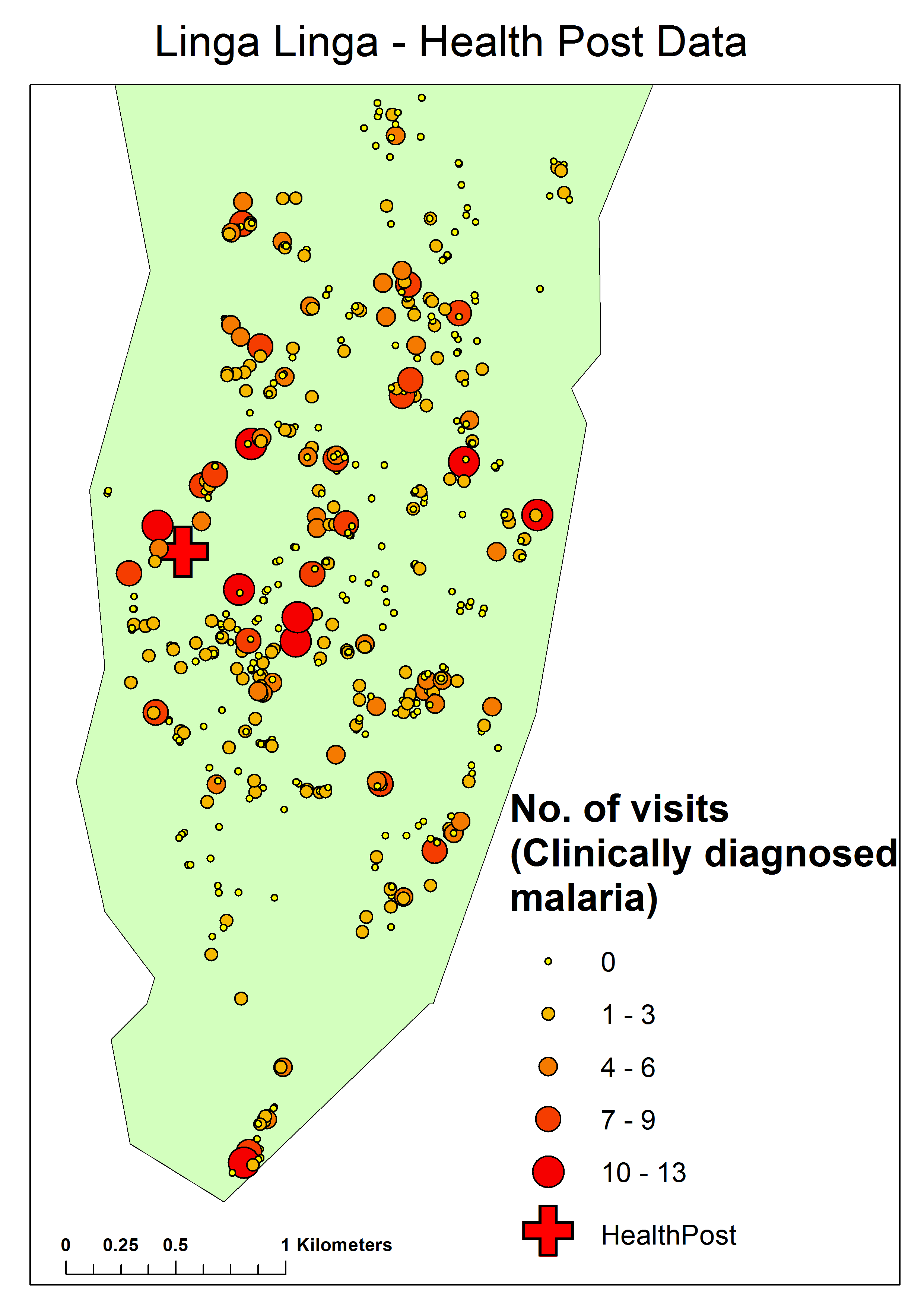
 Figure 3: Number of visits to the health post where malaria was clinically diagnosed


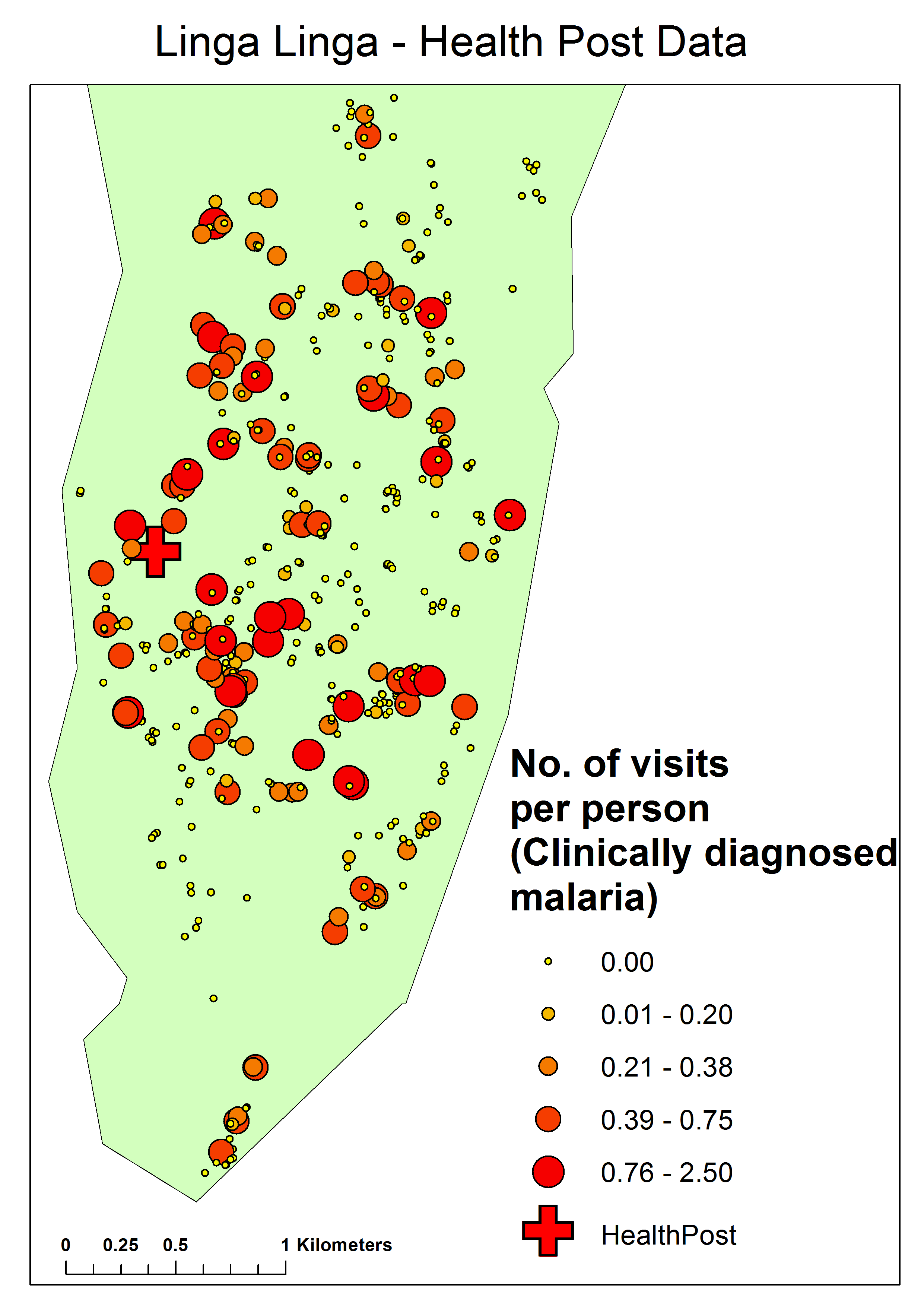


Figure 4: Number of visits to the health post where malaria was clinically diagnosed per person in the household


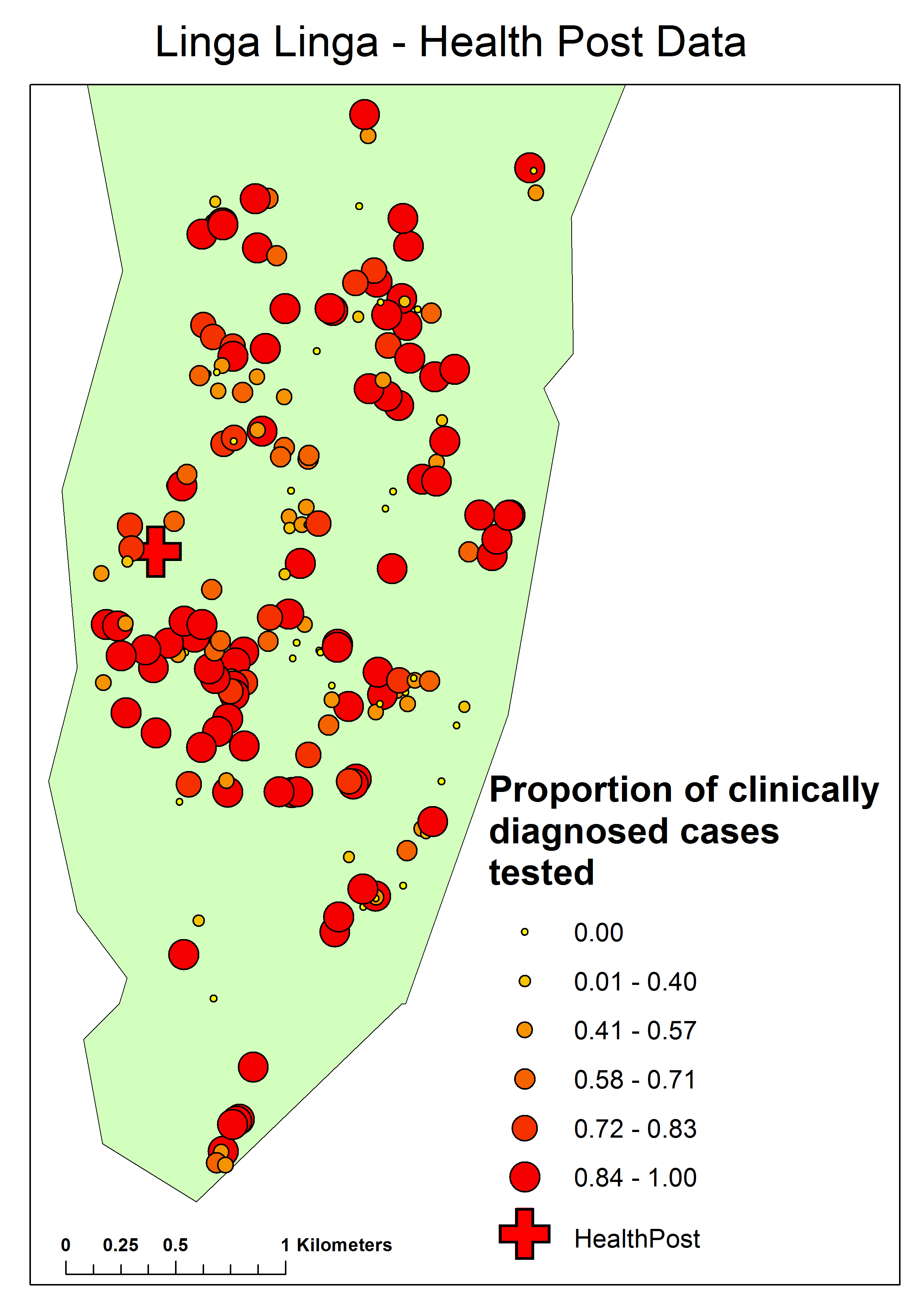


Figure 5: Proportion of visits clinically diagnosed with malaria that were subsequently tested for malaria


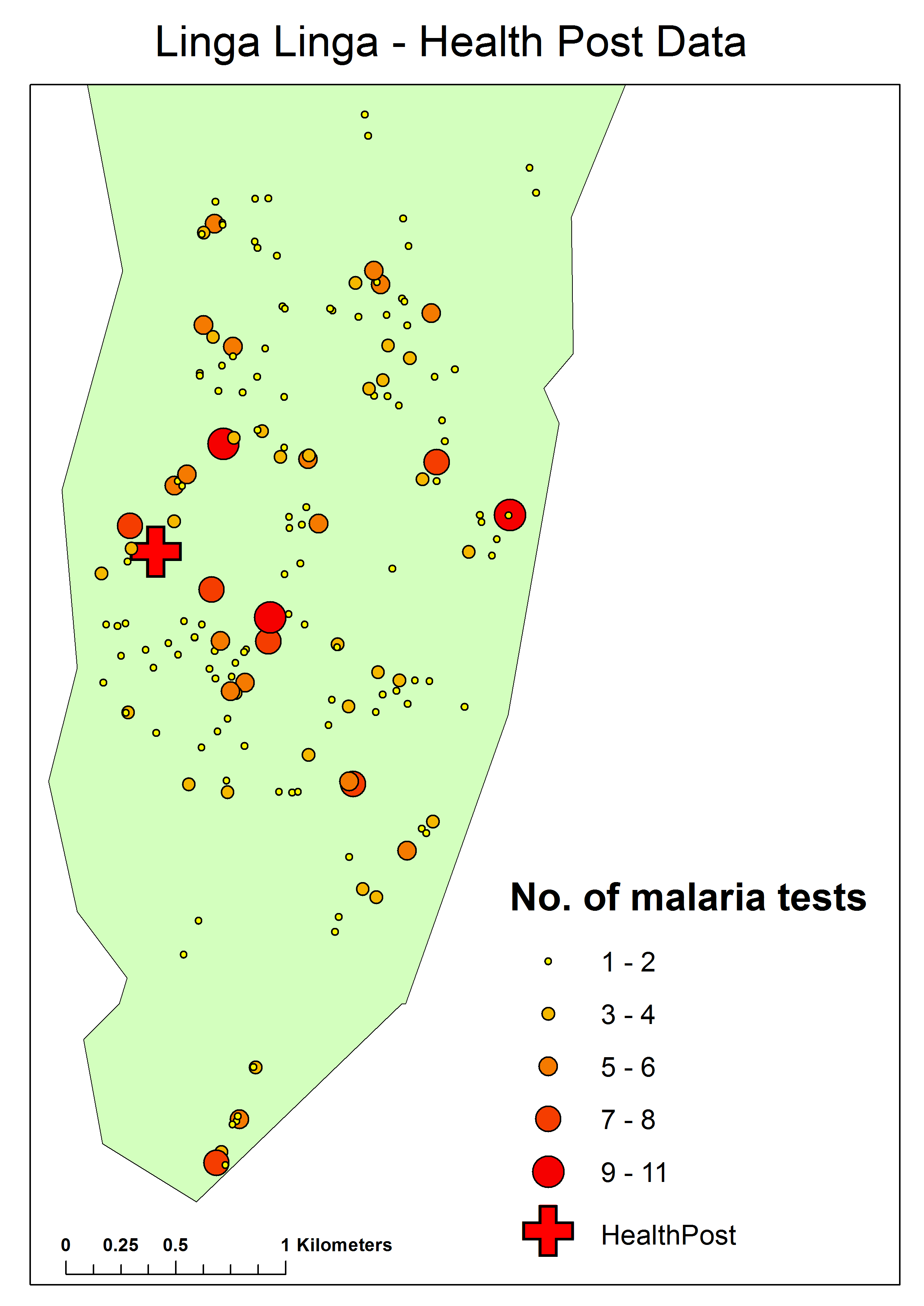
Figure 6: Number of health post visits that were clinically diagnosed with malaria, and subsequently tested for malaria
